# Polymerase Activity, Protein-Protein Interaction, and Cellular Localization of the Usutu Virus NS5 Protein

**DOI:** 10.1128/AAC.01573-19

**Published:** 2019-12-20

**Authors:** L. Albentosa-González, P. Clemente-Casares, R. Sabariegos, A. Mas

**Affiliations:** a Unidad de Medicina Molecular, Centro Regional de Investigaciones Biomédicas, Albacete, Spain; b Facultad de Farmacia, Universidad de Castilla—La Mancha, Albacete, Spain; c Facultad de Medicina, Universidad de Castilla—La Mancha, Albacete, Spain; d Unidad de Biomedicina UCLM-CSIC, Albacete, Spain

**Keywords:** Usutu virus, replicase, NS5, RNA-dependent RNA-polymerase, cooperative activity, subcellular localization, antivirals

## Abstract

Usutu virus (USUV) has become increasingly relevant in recent years, with large outbreaks that sporadically have affected humans being reported in wildlife. Similarly to the rest of flaviviruses, USUV contains a positive-sense single-stranded RNA genome which is replicated by the activity of nonstructural protein 5 (NS5). USUV NS5 shows high sequence identity with the remaining viruses in this genus. This permitted us to identify the predicted methyltransferase domain and the RNA-dependent RNA polymerase domain (RdRpD). Owing to their high degree of conservation, viral polymerases are considered priority targets for the development of antiviral compounds. In the present study, we cloned and expressed the entire NS5 and the RdRpD in a heterologous system and used purified preparations for protein characterizations. We determined the optimal reaction conditions by investigating how variations in different physicochemical parameters, such as buffer concentration, temperature, and pH, affect RNA polymerization activity. We also found that USUV polymerase, but not the full-length NS5, exhibits cooperative activity in the synthesis of RNA and that the RdRp activity is not inhibited by sofosbuvir. To further examine the characteristics of USUV polymerase in a more specifically biological context, we have expressed NS5 and the RdRpD in eukaryotic cells and analyzed their subcellular location. NS5 is predominantly found in the cytoplasm; a significant proportion is directed to the nucleus, and this translocation involves nuclear location signals (NLS) located at least between the MTase and RdRpD domains.

## Abstract (precis)

Usutu virus (USUV) has become increasingly relevant in recent years, with large outbreaks that sporadically have affected humans being reported in wildlife. Similarly to the rest of flaviviruses, USUV contains a positive-sense single-stranded RNA genome which is replicated by the activity of nonstructural protein 5 (NS5). USUV NS5 shows high sequence identity with the remaining viruses in this genus.

## INTRODUCTION

Positive-sense single-stranded RNA [(+)ssRNA] viruses cause >75% of all viral diseases in humans, animals, and plants (1). Usutu virus (USUV) is a (+)ssRNA mosquito-borne arbovirus belonging to the genus *Flavivirus* (2). USUV was first identified in South Africa in 1959, but it was not until the description of the first identified cases of infection in humans, first in Africa (3) and later in Europe (4), that it became better known. It presents great interest, due not only to its pathogenicity for humans but also to its similarity to other emerging arboviruses such as West Nile virus (WNV) and other members of the Japanese encephalitis virus (JEV) complex (5). USUV is mostly transmitted to vertebrate hosts through bites of infected mosquitoes, mainly Culex pipiens. Birds are considered the natural vertebrate hosts, while humans are generally dead-end hosts, supporting viral replication but not viral transmission. In recent years, a growing number of cases of neurological disease have been associated with USUV, underlining its increasing relevance with respect to public health (5). Some key reviews can be found in recently published bibliographies (2, 6).

Similarly to other related flaviviruses, the 5′ end of USUV genome has a methylated nucleotide cap to enable canonical cellular translation initiation. The genomic 3′ end lacks a polyadenylate tract and is organized in secondary structures that form a loop (2). The replicative cycle of USUV has not yet been analyzed in depth, but it appears to be very similar to that of other flaviviruses. An USUV particle enters into the cell through clathrin-mediated endocytosis, after which the viral genome is uncoated and released into the cellular cytoplasm. There, the viral genomic RNA is recognized by the cellular translation machinery, leading to the synthesis of a single polyprotein of around 3,000 amino acids in size. This polypeptide is processed cotranslationally and posttranslationally by host and viral proteases to yield at least 11 mature proteins termed the capsid (C), pr peptide (pr), small envelope protein (M), envelope protein (E), and nonstructural proteins 1 to 5 (NS1, NS2A, NS2B, NS3, NS4A, NS4B, and NS5) (2).

NS5 presents the activity responsible for the replication of the viral genome. It contains a RNA-dependent RNA polymerase domain (RdRpD) and a methyltransferase (MTase) domain, which catalyzes the capping of new viral RNA molecules. Hence, first, the synthesis of new viral RNA chains of negative polarity by NS5, using the viral genome of positive polarity as the template, is required to complete a replication cycle. Then, negative RNA strands serve as templates for subsequent synthesis of novel USUV RNA genomes of positive polarity that are packed into new viral particles and leave the cell by exploiting cellular secretory pathways (2). Both enzymatic activities in flavivirus NS5, i.e., viral RNA synthesis and capping, are currently being investigated as targets for developing novel antiflavivirus drugs (7). Some molecules have already been tested against several members of this virus family (8–10). NS5 interacts with different host proteins to support viral replication and also, in some cases, to interfere with or modulate cellular functions (11). Specifically, it has been documented that NS5 migrates to the nucleus, where it establishes interactions with cellular factors that affect the normal expression of several host genes to favor viral replication. The primary amino acid sequence of the flaviviral NS5 protein has conserved nuclear localization signals (NLS) (12, 13). Although the function of NS5 in the nucleus remains controversial, some authors have proposed the nucleocytoplasmic trafficking of NS5 as a target for drug design (14–19).

Here, we investigated the enzymatic properties of the poorly characterized USUV NS5 protein in different biochemical and cellular studies. Specifically, we established the optimal conditions for USUV RNA synthesis *in vitro* and investigated its subcellular localization after expression in human cells. This information is likely to be highly relevant for the identification and development of NS5 targeting antiviral drugs.

## RESULTS

### NS5 and RdRpD cloning, expression, and purification.

**FIG 1:**
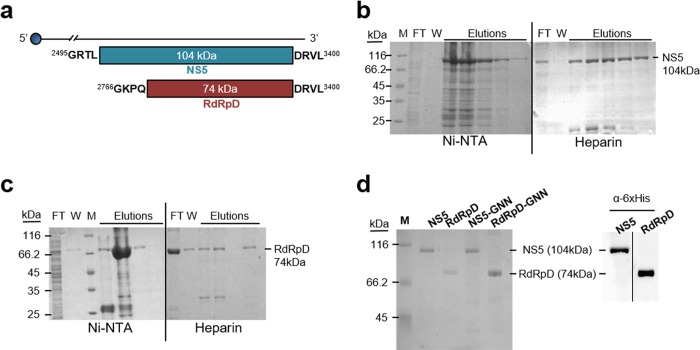
Expression and purification of USUV full-length NS5 and RdRpD. (a) Scheme showing the complete USUV genome with a capped 5′ end (blue circle) and the size of the cloned products as well as the positions and amino acid residues of the N-terminal and C-terminal ends. The numbering refers to USUV strain Vienna 2001 from Austria (GenBank accession number AY453411). (b to d) Protein purification. Bacteria expressing the protein of interest were harvested and lysed as specified in Materials and Methods. Lysates were filtered and loaded onto an affinity chromatography column (Ni-NTA). The aliquots containing the recombinant protein were identified, pooled, and loaded onto an ion exchange chromatography column (Heparin). The elution fractions obtained in each purification step obtained for NS5 (b) and RdRpD (c) were resolved by SDS-PAGE and Coomassie blue staining. M, molecular weight marker; FT, flowthrough; W, washing step. (d) SDS-PAGE (left) and immunoblot using anti-6×His antibodies (right) of proteins isolated after two-step purification. The identity of each protein is indicated at the top of the panel.

DNA fragments spanning residues 2495 to 3400 (numbering refers to USUV strain Vienna 2001 [GenBank accession number AY453411]) encoding the full-length NS5 protein and residues 2766 to 3400 encoding the polymerase catalytic domain (RdRpD) of USUV (Fig. 1a) were amplified by PCR and cloned into pET-21b (Novagen, Madison, WI, USA) and pcDNA3 (Thermo Fisher Scientific) using the forward and reverse primers listed in Table 1. pET-USUV-NS5-F and pET-USUV-R were used to amplify and clone the NS5 fragment into the pET expression vector and pET-USUV-RdRp-F and pET-USUV-R for the RdRpD fragment. The primers used for the amplification of NS5-coding and RdRpD-coding regions and for their cloning into pcDNA3 were primers pcDNA-USUV-NS5-F and pcDNA-USUV-R and primers pcDNA-USUV-RdRp-F and pcDNA-USUV-R, respectively.

**TABLE 1:** Oligonucleotides used in this study

Name	Sequence (5′→3′)^*a*^	5′-end position^*b*^
pET-USUV-NS5-F	GGCGGCTAGCGGAAGACCAGGAGGAAGGAC	7684
pET-USUV-RdRp-F	GGCGGCTAGCGGGAAGCCCCAGCCACATAC	8485
pET-USUV-R	GCGGCTCGAGCAAAACCCTGTCCTCCTGGAC	10398
pcDNA-USUV-NS5-F	AAGCTTGCCATG*TACCCATACGATGTTCCAGATTACGCT*GGAAGACCAGGAGGAAGGAC	7684
pcDNA-USUV-R	CTCGAGTTACAAAACCCTGTCCTCCTGGAC	10398
pcDNA-USUV-RdRp-F	AAGCTTGCCATG*TACCCATACGATGTTCCAGATTACGCT*GGGAAGCCCCAGCCACATAC	8485
USUV-GNN-F	GGCTGTGTGAGTGGAAATAATTGTGTGTCAAGC	9672
USUV-REK-AAA-F	GGGCTTTTCTCGCAGCAGCAGCGAAGCCAAGGTTGTG	8834
USUV-REK-AAA-R	CACAACCTTGGCTTCGCTGCTGCTGCGAGAAAAGCCC	8870
USUV20^*c*^	GCUCACGCAGACGAACGACU	1

a Restriction sites are underlined as follows: NheI for pET forward primers; HindIII for pcDNA forward primers; XhoI for reverse primers. The sequence corresponding to the HA epitope is indicated in italics.

b The numbering refers to USUV strain Vienna 2001 from Austria (GenBank accession number AY453411).

c USUV20 is an RNA molecule.

NS5 protein was concentrated from heparin elutions (Fig. 1b), whereas RdRpD was concentrated from the heparin flowthrough (Fig. 1c). The identity of the full-length NS5 was confirmed by mass spectrometry (data not shown), and both NS5 and RdRpD were detected by immunoblotting using anti-6×His antibodies (Fig. 1d). Lethal mutants were purified following the same protocol. The purified full-length NS5 protein and the RdRpD and the corresponding null-activity mutants NS5-GNN and RdRpD-GNN are shown in Fig. 1d.

### Characterization of RNA polymerase activity in USUV NS5 and RdRpD.

**FIG 2:**
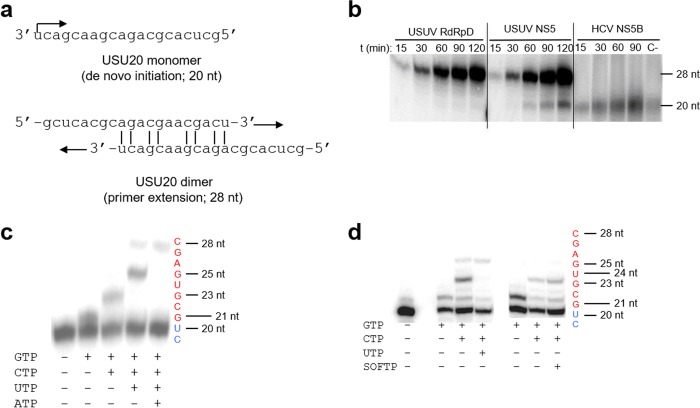
RNA synthesis by USUV full-length NS5 and the polymerase domain. (a) Nucleotide sequence of USU20 RNA as a monomer and a predicted dimeric conformation. The monomeric form as the template gives rise to a product of 20 nucleotides (nt) in length by *de novo* synthesis, while the dimeric form gives rise to a product of 28 nucleotides in length by primer extension synthesis. (b) Polyacrylamide gel showing the products obtained at different time points after incubation of USUV RdRpD, USUV NS5, and HCV NS5B with heteropolymeric template USUV20 (from 15 to 120 min) in the presence of nucleotides and [α-^32^P]CTP. The 20-mer product corresponding to *de novo* synthesis (20 nt) and the primer-extended product (28 nt) are indicated on the right. The “C-” lane corresponds to the USUV20 oligonucleotide labeled at its 5′ end with [γ-^32^P]CTP. (c) Polyacrylamide gel showing the products obtained in the presence of GTP, GTP plus CTP, GTP plus CTP plus UTP, and the four nucleotides after incubation of USUV RdRpD with heteropolymeric template USUV20 previously labeled at its 5′ end with [γ-^32^P]CTP. (d) Polyacrylamide gel showing the products obtained as described for panel c and showing the reaction in the presence of SOFTP. Reactions were carried out during 120 min. The positions of the unextended primer (+20), the GTP-terminated primer (+21 and +23 when CTP was also present in the reaction), the UTP-terminated and SOF-terminated primer (+25), and the fully extended product (+28) are indicated on the right. The product sequence is indicated on the right, with positions +19 and +20 of the oligonucleotide highlighted in blue and newly synthesized extended products in red. All nucleotides were at a final concentration of 100 μM.

The ability of NS5 and RdRpD to utilize heteropolymeric RNA templates was assessed using RNA oligonucleotide USUV20 as the template (Fig. 2a). As a control, hepacivirus C (HCV) NS5B was used. This RdRp synthesizes a 20-mer product corresponding to *de novo* synthesis using USUV20 as the template. USUV NS5 protein and, to a lesser extent, the recombinant RdRpD protein were also competent in synthesizing this 20-mer product (*de novo* synthesis). In addition, both NS5 and RdRpD synthesized larger products, probably as a consequence of primer extension (28 nucleotides [nt]; Fig. 2b). To identify the product of primer extension, the USUV20 oligonucleotide was labeled at the 5′ end, and the reaction was performed as described above but with different combinations of nucleotides. The result (Fig. 2c) confirmed that the primer extension products shown in Fig. 2b represent the consequence of elongation of the dimer that is depicted in Fig. 2a. The presence of *de novo* RNA polymerase activity in the purified proteins was also tested using a poly(rC) homopolymeric template in the absence of primer. Both proteins were able to incorporate [^3^H]-labeled GMP into a poly(rG) product that was synthesized *de novo*. A typical time course of poly(rG) formation is shown in Fig. S1 in the supplemental material.

We also analyzed the inhibition of RdRp activity by sofosbuvir triphosphate (SOFTP) treatment. SOFTP is a competitive nucleoside analogue that has been proved to be active against HCV and other flaviviruses such as dengue virus (DENV) (20). To do this, we elongated the USUV20 labeled at its 5′ end in the presence of GTP alone, of GTP and CTP, and of GTP, CTP, and UTP, all at a concentration of 100 μM, or replaced UTP with SOFTP (also at a final concentration of 100 μM). The results are shown in Fig. 2d, where it is shown that at the concentrations used, the NS5 protein is not able to incorporate SOFTP in the chain of new synthesis. In order to check the activity of SOFTP, we carried out the same experiment using RNA oligonucleotide LE19 labeled at its 5′ end and the HCV polymerase NS5B. The result (Fig. S1b) shows incorporation of SOF (when it was present in the reaction) at both 70 and 700 μM and at similar levels at both concentrations. Therefore, the SOFTP molecule is active and is not incorporated during RNA synthesis by USUV NS5 protein.

### RNA-dependent RNA-polymerase activity conditions.

**FIG 3:**
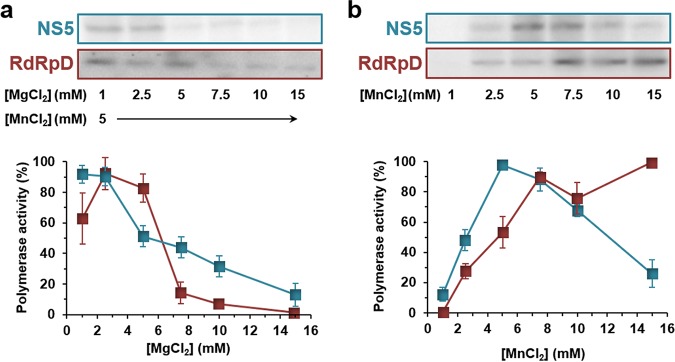
Effect of divalent cations on RdRp activity. Relative levels of RNA polymerase activity for USUV full-length NS5 (cyan) and polymerase domain RdRpD (dark red) in the presence of increasing concentrations of MgCl_2_ (a) or MnCl_2_ (b) as described in Materials and Methods. Data presented in panel a were obtained at a constant concentration of MnCl_2_. A gel representative of each experiment is shown above the corresponding graph. Polymerase activity was normalized to the maximum activity value observed, which was given an arbitrary value of 100%. Nucleotides were at a final concentration of 100 μM. Data represent means (± standard errors of the means [SEM]) of results from at least three independent experiments.

The enzymatic activities of RNA virus polymerases are dependent on different cofactors and environmental conditions. To characterize the optimal reaction conditions, we have investigated how alterations to the polymerase electric charge (ionic strength and pH), the concentration of divalent cations that participate in the polymerase catalytic process, and temperature influence the polymerization process. To define the optimal conditions of the assay for RdRp activity, we carried out experiments in which these variables were analyzed separately. In this optimization assay, we measured the relative increases in the primer extension activity product levels (Fig. 2b). No USUV RNA polymerase activity was observed when magnesium was used only as a divalent cation, in either its acetate or chloride form (Fig. S2). Therefore, experiments were performed with MgCl_2_ supplemented with MnCl_2_ at 5 mM. Optimal polymerase activity was found at magnesium concentrations ranging from 1 to 2.5 mM. Optimal activity was observed at lower MgCl_2_ concentrations for full-length NS5 than for RdRpD (Fig. 3a). The maximum activity observed for full-length NS5 was achieved at 5 mM MnCl_2_ and then decreased gradually as the concentration of manganese increased. RdRpD polymerase activity remained constant at MnCl_2_ concentrations above 7.5 mM MnCl_2_ (Fig. 3b).

**FIG 4:**
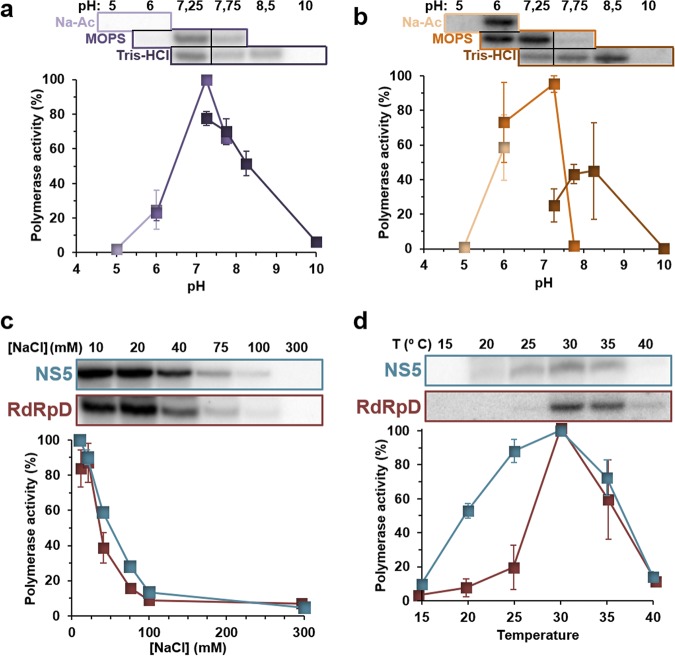
Effect of pH, ionic strength, and temperature on USUV RNA polymerase activity. (a and b) Relative RNA polymerase activity levels of full-length NS5 (a) and RdRpD (b) in reactions carried out at different pH. Different buffers were used for each pH range tested as follows: acetate (lighter colors; pH range of 5 to 6), MOPS (middle-intensity colors; pH range of 6 to 7.75), and Tris-HCl (darkest colors; pH range of 7.25 to 10). (c and d) Relative RNA polymerase activity levels of NS5 (cyan) and RdRpD (dark red) in the presence of increasing concentrations of NaCl (c) and as a function of temperature (d). A gel representative of each experiment is shown above the corresponding graph. Polymerase activity was normalized to the maximum level observed, which was arbitrarily set at a value of 100%. Nucleotides were at a final concentration of 100 μM. Data represent means (± standard errors of the means [SEM]) of results from at least three independent experiments.

We next analyzed how variations in pH levels affected the reaction. To this end, we used three different buffers, acetate (pH 5 to 6), MOPS (morpholinepropanesulfonic acid; pH 6 to 7.75), and Tris-HCl (pH 7.25 to 10), which allowed us to cover the entire pH scale from 5 to 10. Optimum activity was observed at pH 7.25, and no major differences were observed between full-length NS5 (Fig. 4a) and RdRpD (Fig. 4b) proteins. NS5 showed slightly better activity at higher pH values, while RdRpD tended to show higher activity at lower pH values. Reactions at pH values below 5 or above 10 rendered undetectable polymerization products for both proteins (Fig. 4; parallel experiments). Interestingly, the activity of RdRpD was more sensitive to buffer changes in the pH range between 7 and 8 than the activity of full-length NS5 (compare Fig. 4a and b). We then analyzed the effect of ionic strength on the polymerase activity. For both full-length NS5 and RdRpD proteins, RNA polymerase activity decreased as the buffer ionic strength increased, with activity values below 30% for NaCl concentrations equal to or higher than 75 mM (Fig. 4c). Full-length NS5 was slightly more active than RdRpD in the range of 20 to 100 mM NaCl, suggesting that changes in the electric charge have a larger impact on RdRpD than on full-length NS5 protein.

To determine the optimal temperature for the reaction, we measured polymerase activity in the range of 15°C to 40°C. We found optimal activities at 30°C for both proteins (Fig. 4d). Nevertheless, we observed significant differences between NS5 and RdRpD at lower temperatures, with relative activity levels of >50% for NS5 between 20°C and 25°C, while RdRpD showed limited activity within this temperature range (Fig. 4d).

### USUV RNA polymerase displays cooperative activity.

**FIG 5:**
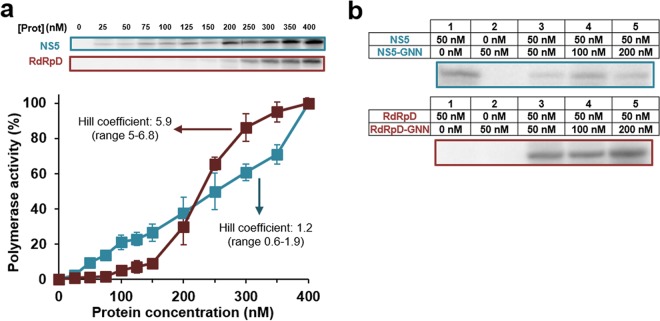
RNA polymerase activity is cooperative. (a) Relative RNA polymerase activity levels of NS5 (cyan) and RdRpD (dark red) in the presence of increasing concentrations of each protein as described in Materials and Methods. A representative gel for each graphic representation is shown in the upper part of the panel. Polymerase activity was normalized to the maximum level, which was arbitrarily set to a value of 100%. Data represent means (± standard errors of the means [SEM]) of results from at least three independent experiments. (b) Effect of in *trans* complementation performed using a lethal RdRp mutant for analysis of polymerase activity. Data from NS5 and RdRpD experiments are shown in the upper and lower panels, respectively. Concentrations of NS5, RdRpD, and their corresponding lethal mutants are shown. Nucleotides were at a final concentration of 100 μM.

It has been reported previously that some viral polymerases can act cooperatively (21–23). To examine whether USUV NS5 is a cooperative polymerase, we determined the amount of product that is synthesized at increasing protein concentrations. While full-length NS5 protein showed a nearly linear increase in activity (Fig. 5a), RdRpD activity followed an sigmoidal pattern, with very limited activity detected at concentrations of <150 nM followed by a rapid increase between 150 and 200 nM, where the levels reached were similar to those seen with full-length NS5 (Fig. 5a). The Hill coefficient values extrapolated from these curves were 1.2 (range, 0.6 to 1.9) and 5.9 (5 to 6.8) for full-length NS5 and RdRpD proteins, respectively.

To further investigate the functional significance of USUV oligomerization, NS5 and RdRpD activities were monitored in the presence of increasing concentrations of their inactive mutant counterparts. Lethal mutants of NS5 (NS5-GNN) and RdRpD (RdRpD-GNN) were obtained by replacing the catalytic triad GDD in motif C by GNN. Neither of the two mutants isolated showed any residual polymerase activity (Fig. 5b, lane 2; see also Fig. S1c). After setting up the conditions in several preliminary experiments, we performed reactions where we complemented our catalytically active wild-type polymerases (NS5 or RdRpD constant concentration of 50 nM) with increasing amounts of the corresponding inactive partner (NS5-GNN or RdRpD-GNN). In the absence of lethal mutants, the activity of each wild-type polymerase was barely detectable, although NS5 was slightly more active than the RdRpD protein (Fig. 5a and b [lane 1]). When increasing amounts of catalytically inactive mutant were added to 50 nM RdRpD wild-type enzyme, we observed significant increases in activity (Fig. 5b, lanes 3 to 5). We found that even at a 1:1 ratio of mutant/wild-type RdRpD (50 nM GNN mutant), RNA synthesis was significantly stimulated (Fig. 5b).

### Subcellular localization of USUV NS5 and RdRpD.

**FIG 6:**
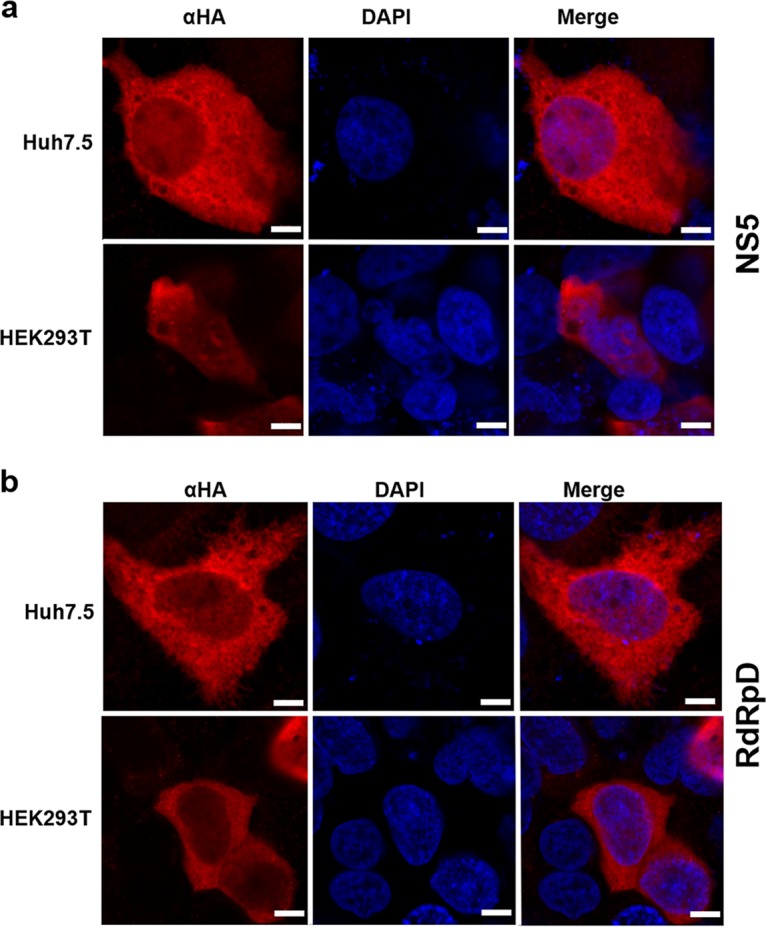
Subcellular localization of NS5 and RdRpD. Huh7.5 and HEK293T cells were transfected with transient expression plasmids encoding HA-tagged versions of either NS5 (a) or RdRpD (b) (pcDNA3-NS5-HA or pcDNA3-RdRpD-HA). Immunocytochemistry was performed using antibodies against HA (in red) and with DAPI (in blue) to detect cell nuclei. αHA panels correspond to immunostaining with anti-HA, whereas DAPI panels correspond to staining of cell nuclei. Merged images show an overlay with colocalization. Scale bars correspond to 5 μm.

**FIG 7:**
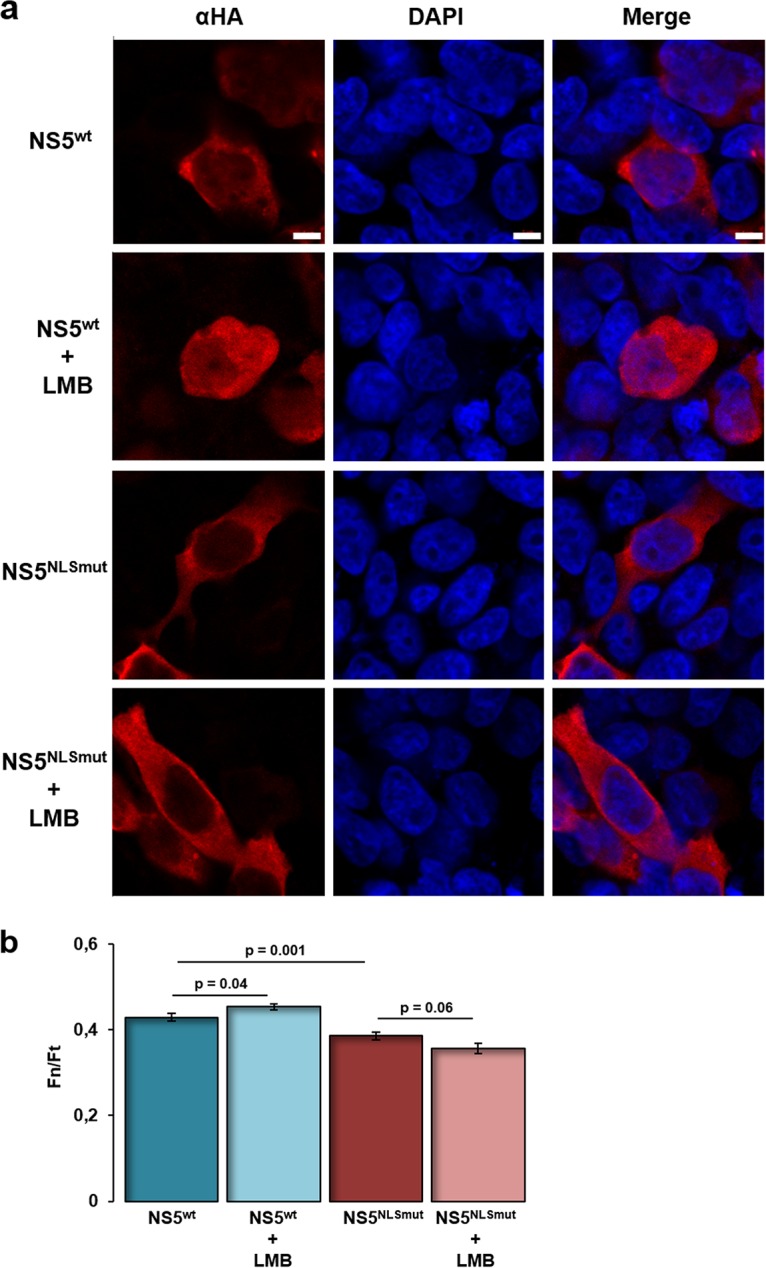
Role of NLS in the subcellular localization of USUV NS5. (a) HEK293T cells were transfected with a transient expression plasmid encoding full-length NS5 (pcDNA3-NS5-HA) or the corresponding NLS mutant containing amino acid changes REK to AAA (pcDNA3-NS5^NLSmut^-HA). When indicated, cellular supernatants were removed at 24 h posttransfection and 40 nM LMB in complete media was added to the cells. At 30 h posttransfection, cells were fixed for immunofluorescence. Immunocytochemistry was performed using antibodies against HA (in red) to detect NS5 or NS5^NLSmut^ and with DAPI (in blue) to detect cell nuclei. αHA panels correspond to immunostaining with anti-HA, whereas DAPI panels correspond to staining of cell nuclei. Merged images show an overlay with colocalization. Scale bars correspond to 5 μm. (b) Mean nuclear fluorescence was quantified using Zeiss ZEN 2 software (blue edition), and Fn/Ft values were determined. Twenty cells per sample were analyzed from independent duplicate experiments. Data represent means (± standard errors of the means [SEM]). *P* values are indicated above.

To gain a broader understanding of USUV replicase activities in the cellular context, we decided to investigate the subcellular localization of both full-length NS5 (Fig. 6a) and RdRpD (Fig. 6b). In both cases, these proteins were preferentially located in the cytoplasmic region regardless of whether they were expressed in Huh7.5 or HEK293T cells (Fig. 6). Nonetheless, a significant amount of USUV polymerase was found in the nucleus. Flavivirus NS5 has several conserved nuclear localization signals (NLS), mostly in the linker region between the MTase and RdRp domains. To test whether these predicted NLS are relevant to the nuclear localization of USUV NS5, we mutated one of them (Fig. S3; homologue to mutant KKK 387–389 reported in reference 14) and analyzed changes in fluorescence intensity in the nuclei by confocal microscopy. The wild-type and mutant proteins were found to be expressed at similar levels (Fig. S4c). Nuclear signal intensity (measured as a Fn/Ft ratio) decreased slightly although significantly in NS5^NLSmut^-transfected HEK293T cells (Fig. 7), as well as in Huh7.5 cells (Fig. S4). Both cell lines were then transfected with wild-type NLS (NS5^wt^) or NLS mutant (NS5^NLSmut^) and treated with a nuclear export inhibitor (leptomycin B [LMB]), as described in Materials and Methods. No statistically significant differences in the nuclear signal were observed in cells expressing NS5^wt^ or NS5^NLSmut^ after treatment with this inhibitor (Fig. 7; see also Fig. S4).

## DISCUSSION

Flaviviral NS5 protein is a major therapeutic target for the development of antiviral compounds due to the centrality of its activity in the virus life cycle and its high degree of conservation among different viruses in this genus (see Fig. S3 in the supplemental material). In this work, we cloned, expressed, purified, and characterized the experimental conditions under which USUV NS5 polymerase activity is optimal. We have also identified the subcellular regions where NS5 localizes during expression in the cell and identified a putative signal for nuclear transport.

USUV NS5 is predicted to contain the priming loop structure (amino acids 3284 to 3309 [Vienna2001 strain; GenBank accession number AY453411]) (Fig. S3). In other related flaviviruses, this motif is responsible for the initiation of replication in the absence of primer (24). The identification of this conserved motif in the primary amino acid sequence of USUV NS5 and the results shown in Fig. 2 (see also Fig. S1a) suggest that RNA synthesis initiation occurs in the absence of primer (*de novo* activity) (25–29), as previously described for other flaviviral polymerases (30). In addition, and as has already been described for the NS5 protein of the dengue virus (20), the concentration of SOFTP necessary to achieve the incorporation of this drug in 50% of the RNA that is elongating is higher than 100 μM (Fig. 2d). *In vivo* studies are needed to find out if this drug is really effective in USUV infection.

Nucleic acid polymerases use divalent cations to stabilize the reaction intermediates and favor nucleophilic attack and phosphodiester bond formation during viral RNA synthesis (31). Although both magnesium and manganese cations function as competent divalent cations *in vitro*, it is believed that magnesium cation is used mostly in physiological environments (30). USUV NS5 and RdRpD use both Mg^2+^ and Mn^2+^ as catalytic ions (Fig. 3). No major differences were found between full-length protein and RdRpD in their requirements for these molecules. Variations in polymerase activity as a function of their concentration were similar to those previously described for DENV and WNV RdRpD (30) and HCV NS5B (32). As documented for DENV and WNV polymerase activities in the presence of homopolymer templates (30), Mg^2+^ had to be supplemented with Mn^2+^ to detect activity. Overall, full-length NS5 protein was more active than RdRpD under most of the conditions analyzed. NS5 protein showed higher activity at low MgCl_2_ or NaCl concentrations, in the presence of different buffers, and at pH values between 7 and 8 (Fig. 3 and 4). These data may indicate that full-length NS5 protein could be more stable than the corresponding RdRp domain alone. The proposed interactions between the MTase and RdRp domains could be responsible for this possible gain in NS5 stability (25, 29, 33–36). The impact of alterations affecting interdomain interactions is currently being analyzed in the context of *in vivo* replication and as a potential target for the design of antiviral drugs (33–35).

Electrostatic interactions are very important during the catalytic cycle of nucleic acid polymerases (31). We have obtained peaks of maximum activity at pH 7.25 for both full-length and RdRpD proteins (Fig. 4a and b). This is in agreement with data previously described for HCV NS5B protein (21, 37). We have also analyzed the effect of ionic strength, and both the full-length protein and the RdRpD are very sensitive to increases in NaCl concentrations (Fig. 4c). Elevated ionic forces are typically required during the process of flavivirus polymerase purification to avoid protein aggregation and precipitation. However, for different (+)ssRNA viruses, these protein-protein interactions can be important for RNA synthesis (21, 23). Our experiments (Fig. 5) have confirmed that RdRpD is highly cooperative whereas full-length NS5 protein is less cooperative (Hill coefficient values of 5.9 and 1.2, respectively). These results were confirmed by in *trans* complementation experiments using inactive variants containing lethal mutations (NS5-GNN and RdRpD-GNN). Previous studies suggested that the MTase and RdRp domains initially existed as two separate proteins that then fused to form flavivirus NS5. This NS5 protein may have evolved in divergent manners that led to the two major conformations described to date (i.e., those of DENV and JEV) (25). This evolutionary mechanism would also explain why the RdRpD domain conserved the oligomerizing capacity present in hepacivirus (NS5B of HCV) (37, 38) and picornavirus polymerases (23). Full-length NS5 showed a low Hill coefficient and was not complemented by NS5-GNN. Thus, our results support the idea that USUV is similar to DENV and Zika virus (ZIKV) polymerases, and previous structural data suggest that their NS5 polymerases form homodimers (25, 28, 39–41).

Flavivirus full-length NS5 is found not only in the cytoplasm but also in the nucleus (11, 12, 15, 17, 42). USUV is not an exception, and here we found signal of both USUV NS5 and RdRpD in the cytoplasm as well as in the nucleus (Fig. 6). We also propose a possible role in nuclear internalization for one of the putative NLS identified in the USUV NS5 protein sequence (Fig. 7; see also Fig. S4). The relevance of NLS and nuclear localization of NS5 to USUV replication and/or virus-mediated alterations of the host cell remains to be explored.

Owing to its major role in viral replication, the USUV NS5 protein constitutes one of the most important targets for antiviral drug design. It appears that the incidence of human cases of USUV infection is increasing, with a growing number of cases leading to severe neurological disorders similar to those observed for WNV infections (5). In this work, we describe for the first time the main features of full-length USUV NS5, including its polymerase activity as well as important protein-protein interactions and subcellular localization. These data will have great impact on the design of antiviral strategies against this virus.

## MATERIALS AND METHODS

### DNA amplification by PCR and cloning.

USUV coding sequences corresponding to the whole NS5 protein and RdRp domain (RdRpD) were amplified by PCR, using specifically designed primers (Table 1) and PfuTurbo DNA polymerase (Agilent Technologies), with a full-length USUV cDNA (kindly provided by Armando Arias) used as the template. The NS5 and RdRpD fragments were cloned into eukaryotic (pcDNA3) and bacterial (pET21b) expression vectors by employing the restriction enzyme sites included in the primers. Hemagglutinin (HA) epitopes were incorporated in the forward primers of the pcDNA3 constructs (Table 1). The constructs in pET21b were designed to contain 6×His tags at their C termini (included in the vector). Inactive variants of both proteins (NS5-GNN and RdRpD-GNN [GDD in motif C substituted for GNN; see Fig. S3 in the supplemental material]) were obtained by site-directed mutagenesis using a QuikChange Lightning site-directed mutagenesis kit (Agilent Technologies) and NS5 and RdRp pET21b constructs as the templates. A NLS triple-alanine mutant (mutation of R^390^E^391^K^392^ to A^390^A^391^A^392^ positions in Fig. S3 DENV2 sequence [14]) was obtained by site-directed mutagenesis using the NS5 pcDNA3 construct as the template. Primers for mutagenesis are listed in Table 1. All constructs were confirmed by DNA sequencing.

### USUV NS5 and RdRpD protein purification.

pET21 plasmid constructs were used to transform Escherichia coli strain BL21(DE3)pLysS Rosetta. A preinoculum from one positive colony was prepared in 10 ml of LB medium supplemented with ampicillin (Amp; 100 μg/ml) and chloramphenicol (Cm; 17 μg/ml). After 16 h of incubation at 37°C with shaking, a flask containing 500 ml of LB supplemented with the same antibiotics was inoculated with the preinoculum. IPTG (isopropyl-β-d-thiogalactopyranoside; 100 μM final concentration) and absolute ethanol (2% [vol/vol] final concentration) were added when the culture reached an optical density at 600 nm (OD_600_) of between 0.6 and 0.8. Cells were then incubated for an additional 2 h at 4°C, followed by 16 h at 17°C with shaking. The cells were subsequently collected by centrifugation at 5,000 × *g* for 10 min. The sediment was resuspended in 30 ml of buffer A (20 mM Tris-HCl [pH 7], 1 M NaCl, 10% glycerol, 1% Triton X-100, 10 mM imidazole, 1 mM β-mercaptoethanol) supplemented with 1 mM phenylmethylsulfonyl fluoride (PSMF), 1 mg/ml lysozyme, and 1 μg/ml DNase. Cells were then sonicated (20 cycles of 10 s), and the soluble fraction was isolated by centrifugation at 45,000 × *g* for 45 min at 4°C.

The supernatant was filtered using a Steriflip vacuum-driven filtration system (Millipore). The filtrate was then subjected to affinity column chromatography using cross-linked agarose derivatized with nickel-nitrilotriacetic acid (NTA) resin (Thermo Fisher Scientific) that was equilibrated with 20 column volumes (CV) of buffer A. After loading of the sample, the column was washed with 20 CV of buffer A, and the bound protein was eluted by applying two CV of elution buffer (buffer A supplemented with 250 mM Imidazole) five times.

A second chromatographic purification step with heparin Sepharose 6 Fast Flow resin (GE Healthcare) was carried out. To this end, the sample was first diluted in dilution buffer (20 mM Tris-HCl [pH 7.0], 10% glycerol, 1 mM β-mercaptoethanol) to reduce the NaCl concentration to 250 mM. The resin (200 μl) was equilibrated with 7.5 CV of buffer B (20 mM Tris [pH 7], 250 mM NaCl, 10% glycerol, 1 mM β-mercaptoethanol). The sample and the resin were then mixed, incubated for 30 min at 4°C in a Ferris wheel, loaded into a column holder, and washed with 25 CV of buffer B (20 mM Tris-HCl [pH 7], 250 mM NaCl, 10% glycerol, 1 mM β-mercaptoethanol). Five elution fractions (2 CV each) were made with elution buffer (20 mM Tris-HCl [pH 7], 700 mM NaCl, 10% glycerol, 1 mM β-mercaptoethanol). Purified proteins were concentrated using Amicon Ultra 50 K centrifugal filters (Millipore). The aliquots showing the purest proteins and highest concentrations were adjusted to 50% glycerol and stored at –80°C. All purification steps were followed by SDS-PAGE and Coomassie blue staining, and the amount of NS5 or RdRpD was quantified by Bradford protein assay and SDS-PAGE gel imaging. HCV NS5B protein was purified as previously described (21, 37).

### *In vitro* RdRp activity assays.

Polymerase activity tests were performed using a RNA substrate mimicking the 3′ end of the negative strand of the USUV genome (USUV20, Table 1). The conditions for the standard polymerase activity assay were established as follows. A reaction premix was prepared, containing reaction buffer (20 mM MOPS [pH 7.25], 5 mM MnCl_2_, 40 mM NaCl), 300 nM USUV20 RNA (Fig. 2A; see also Table 1), 500 μM GTP (second initiating nucleotide), and 100 nM USUV protein. This mixture was incubated for 15 min at room temperature (RT) to allow NS5/RdRpD complex formation with the RNA template. Then, the reaction was initiated by addition of 100 μM ATP (the first initiating nucleotide), 100 μM UTP, and 1 μCi [α-^32^P]CTP (PerkinElmer Life Sciences) (3,000 Ci mmol). Assays to examine cooperativity were performed in the presence of a 50 nM concentration of each wild-type enzyme supplemented with increasing concentrations of the corresponding inactive mutant. Primer extension activity assays were performed with USUV20 or LE19 oligonucleotides labeled at the 5′ end with [γ-^32^P]ATP and T4 polynucleotide kinase (Promega). Reaction mixtures were incubated under the conditions indicated in each experiment, stopped by adding EDTA/formamide loading buffer, and resolved in a denaturing 23% polyacrylamide–7 M urea gel. Finally, gels were exposed to phosphorimager screens and scanned in a Typhoon 9600 phosphorimager (Molecular Dynamics). The intensity of each band was quantified with ImageLab 6.0 software (Bio-Rad).

Experiments to detect *de novo* synthesis were performed in the presence of 20 mM MOPS (pH 7.25), 5 mM MnCl_2_, poly(rC) 40 ng/μl, 125 μM GTP, 0.5 μCi [^3^H-GTP] (Amersham), and 400 nM protein. The reaction mixture was incubated at 30°C for 2 h and stopped by addition of 150 mM EDTA. Samples were transferred to a DE81 filter (Whatman International Ltd.), which was subsequently washed with 9 ml Na_2_HPO_4_, 9 ml H_2_O, and 3 ml ethanol. The filter was dried at 55°C for 15 min, and radioactive counts were monitored using a model LS6500 scintillation counter (Beckman Coulter).

### Cell culture, Western blotting, immunocytochemistry, and confocal microscopy.

The pcDNA3 constructs obtained for expression in mammalian cells were tested in HEK293-T and Huh-7.5 cell lines. Cells were collected in lysis buffer (100 mM HEPES [pH 7.5], 50 mM NaCl, 0.1% Triton X-100, 5 mM EDTA, 0.125 M EGTA) supplemented with protease inhibitors (Sigma-Aldrich). Protein quantification from cellular lysates was performed by using a bicinchoninic acid (BCA) protein assay kit (Thermo Fisher) following the manufacturer’s instructions. Protein (recombinant or lysates) was loaded onto SDS-PAGE at the appropriate percentage, transferred to polyvinylidene difluoride (PVDF) membranes with a semidry Pierce Power Blot (Thermo Fisher), and blotted against different proteins using specific antibodies. Antibody detection was achieved by the use of enhanced chemiluminescence (Amersham) in a LAS-4000 system (Fujifilm). The blot shown is representative of three with nearly identical results. Tubulin was used as a loading control.

To analyze the subcellular localization of the protein of interest, 2 × 10^5^ cells were seeded in 60-mm-diameter plates in the presence of Dulbecco’s modified Eagle’s medium (DMEM) supplemented with glutamine. The constructs were transfected with Lipofectamine 2000 (Thermo Fisher Scientific). At 24 h posttransfection, cells were fixed to the slide with 2% paraformaldehyde (for 1 h at RT), washed three times with 1× phosphate-buffered saline (PBS), and incubated with a permeabilization and blocking solution containing 5% bovine serum albumin (BSA)–0.3% Triton X-100–PBS for 1 h at RT. Slides were incubated overnight with primary anti-HA antibody (BioLegend). After this, slides were rinsed three times with 1× PBS, secondary antibody (Alexa Fluor 546; Thermo Fisher Scientific) was added to the slides, and the reaction mixture was incubated for 1 h at RT. Unbound secondary antibody was removed by washing slides three times in 1× PBS. Nuclei were stained by incubating the slides with DAPI (4′,6-diamidino-2-phenylindole) for 5 min at RT. After three washing steps were performed with 1× PBS for 5 min each step, cells were mounted with Dako. The samples were analyzed with a Zeiss LSM 710 confocal microscope. Images (8-bit depth; 1,024-by-1,024 format) were acquired using an oil immersion 40× Plan-Neofluar 1.3-numerical aperture (NA) objective, resulting in a pixel size of 208 nm. Two channels were sequentially registered in order to capture Alexa568 (channel 1) and DAPI (channel 2) signals, the first using a 543-nm laser source and collecting 554-to-607-nm wavelength light and the second using a 405-nm laser source and collecting 427-to-494-nm wavelength light. The pinhole was set to obtain 1-μm sections in both channels.

Nuclear translocation was evaluated by measuring the relationship between the brightness intensities of the NS5 signal in the nucleus and the whole cell. Twenty transfected cells showing no signs of condition-altered morphology were selected. The relationship between the signal of NS5 in the whole nucleus (Fn) and the total signal of NS5 (Ft; nucleus plus cytoplasm) was obtained by dividing the total intensity of the signal in channel 1 in the whole nucleus by the intensity measured for the whole cell.

Where indicated, 2 × 10^5^ cells were seeded and transfected and, at 24 h posttransfection, the medium was replaced by complete DMEM containing 40 nM leptomycin B from *Streptomyces* sp. (LMB; Sigma). After 6 h of treatment, cells were fixed in 2% paraformaldehyde, and immunocytochemistry was performed as described above.

### Statistical analyses.

Statistical comparisons among groups were performed using Student's *t* test. Significant differences (*P* values of ≤0.05) between groups are indicated. *P* values of ≤0.01 are considered highly significant.

## Supplementary Material

Supplemental file 1
