# Supplementary material for: Polymerase Activity, Protein-Protein Interaction, and Cellular Localization of the Usutu Virus NS5 Protein
Source: Antimicrob Agents Chemother. 2019 Dec 20;64(1):e01573-19. doi: 10.1128/AAC.01573-19 (PMC7187600; doi:10.1128/AAC.01573-19)
Supplement: Supplemental file 1 [file AAC.01573-19-s0001.pdf]

Supplementary Figure 1a

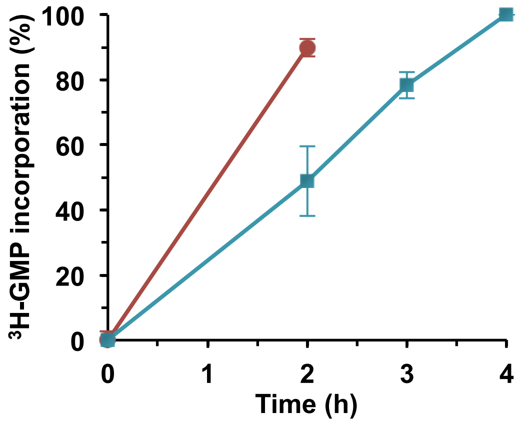

Supplementary figure 1a. 3H-GMP incorporation into a poly(rG) by USUV NS5 and RdRpD. Representation of the RNA polymerase activity product amount after incubation of USUV NS5 (cyan) or RdRpD (dark red) with homopolymeric template poly(C) and [3H]GTP at different time points. Polymerase activity was normalized with respect to its maximum activity that arbitrarily was set at 100. Values represent the means ± standard error of the mean of at least three independent experiments.

Supplementary Figure 1b

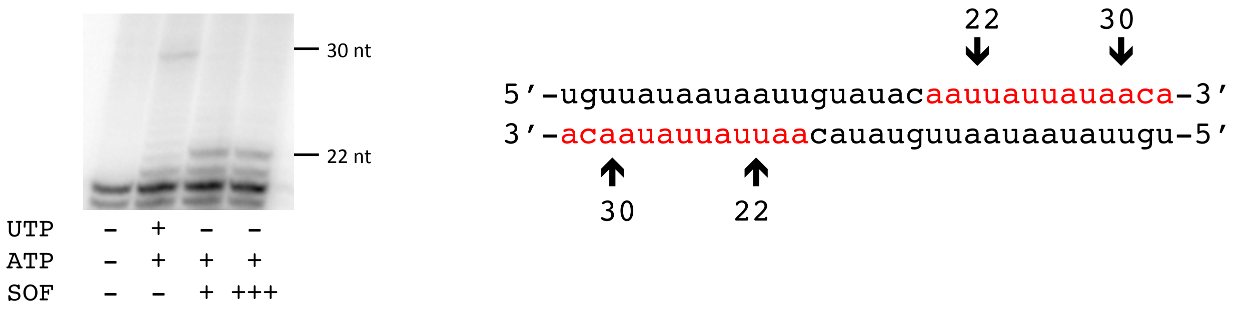

Supplementary figure 1b. Sofosbuvir incorporation into a heteropolymen.ric template:primer by HCV NS5B. LE19 RNA was used for a primer extension assay (in the absence of GTP) to analyze the ability of NS5B HCV polymerase to incorporate Sofosbuvir triphosphate (Left). In the presence of UTP and ATP, NS5B was able to synthesize a 30 nt product. In the presence of ATP and Sofosbuvir (+, 70 **µ**M; +++, 700 **µ**M) NS5B synthesis was stopped rendering a product with the expected size (22 nt). (Right) Template:primer sequence (in black) and expected newly synthesized product sequences (in red) for primer extension reaction are shown. The expected stops at 22 nt (SOF incorporation) and 30 nt (stop because there is no CTP in the reaction) are indicated.

Supplementary Figure 1c

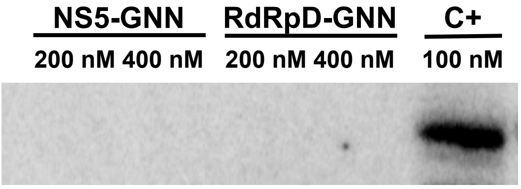

Supplementary figure 1c. RdRp activity of lethal mutants (NS5-GNN and RdRpD-GNN). USUV20 RNA was used for a primer extension assay to analyze the ability of USUV NS5-GNN (left) and RdRpD-GNN (right) lethal mutants to incorporate NTPs. Mutants final concentration were 200 nM and 400 nM. A reaction with $NS5^{wt}$ was carried out using 100 nM enzyme concentration as a positive control. All reactions were carried out for 2 hours at room temperature.

Supplementary Figure 2

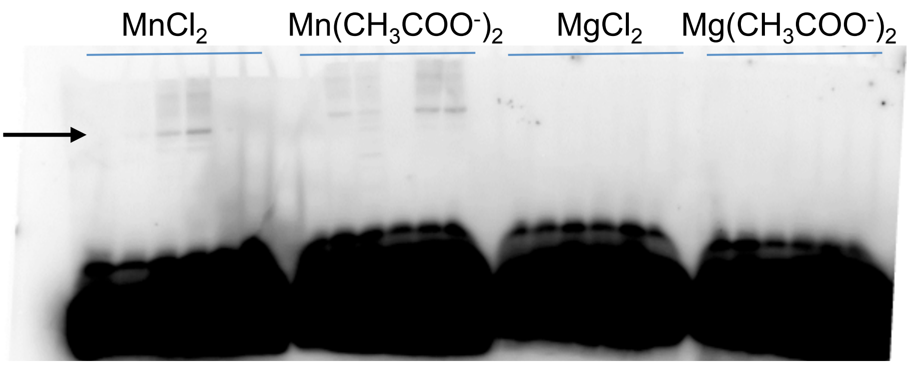

Supplementary figure 2. Divalent cations use. Representative experiment of RNA synthesis by USUV NS5 in the presence of increasing concentrations (1; 2.5; 5; 7.5; 10; 15 mM) of $MnCl_2$, $Mn(CH_3COO^-)_2$, $MgCl_2$ or $Mg(CH_3COO^-)_2$. Only products in the presence of manganese were detected (black arrow).

Supplementary Figure 3

```
USUV     RGRPGGRTLGEQWKEKLNGLSKEDFLKYRKEAITEVDRSAARKARRDGNKTGGHPVSRGSAKLRWMVERQFVKPIGKVVD     80
WNV      RGGAKGRTLGEVWKERLNHMTKEEFTRYRKEAITEVDRSAAKHARREGNITGGHPVSRGTAKLRWLVERRFLEPVGKVVD     80
JEV      RGRPGGRTLGEQWKEKLNAMSREEFFKYRREAIIEVDRTEARRARRENNIVGGHPVSRGSAKLRWLVEKGFVSPIGKVID     80
DENV1    GTGAQGETLGEKWKRQLNQLSKSEFNTYKRSGIIEVDRSEAKEGLKRGE-TTKHAVSRGTAKLRWFVERNLVKPEGKVID     79
DENV2    GTGNIGETLGEKWKSRLNALGKSEFQIYKKSGIQEVDRTLAKEGIKRGE-TDHHAVSRGSAKLRWFVERNMVTPEGKVVD     79
              *.**** ** :** : :.:*  *::..* ****: *:.. : .: .  * ****:*****:**: :: * ***:*

USUV     LGCGRGGWSYYAATLKGVQEVRGYTKGGPGHEEPMLMQSYGWNLVTMKSGVDVYYKPSEPCDTLFCDIGESSSSAEVEEQ     160
WNV      LGCGRGGWCYYMATQKRVQEVKGYTKGGPGHEEPQLVQSYGWNIVTMKSGVDVFYRPSEASDTLLCDIGESSSSAEVEEH     160
JEV      LGCGRGGWSYYAATLKKVQEVRGYTKGGAGHEEPMLMQSYGRNLVSLKSGVDVFYKPSEPSDTLFCDIGESSPSPEVEEQ     160
DENV1    LGCGRGGWSYYCAGLKKVTEVKGYTKGGPGHEEPIPMATYGWNLVKLYSGKDVFFTPPEKCDTLLCDIGESSPNPTIEEG     159
DENV2    LGCGRGGWSYYCGGLKNVREVKGLTKGGPGHEEPIPMSTYGWNLVRLQSGVDVFFIPPEKCDTLLCDIGESSPNPTVEAG     159
         ********.** .  * * **:* **** *****  : :** *:* : ** **:: * * .***:******* .  :*

USUV     RTLRILEMVSDWLQRGPREFCIKVLCPYMPRVMERLEVLQRRYGGGLVRVPLSRNSNHEMYWVSGAAGNIVHAVNMTSQV     240
WNV      RTVRVLEMVEDWLHRGPKEFCIKVLCPYMPKVIEKMETLQRRYGGGLIRNPLSRNSTHEMYWVSHASGNIVHSVNMTSQV     240
JEV      RTLRVLEMTSDWLHRGPREFCIKVLCPYMPKVIEKMEVLQRRFGGGLVRLPLSRNSNHEMYWVSGAAGNVVHAVNMTSQV     240
DENV1    RTLRVLKMVEPWLR-G-NQFCIKILNPYMPSVVETLEQMQRKHGGMLVRNPLSRNSTHEMYWVSCGTGNIVSAVNMTSRM     237
DENV2    RTLRVLNLVENWLN-NNTQFCIKVLNPYMPSVIEKMEALQRKYGGALVRNPLSRNSTHEMYWVSNASGNIVSSVNMISRM     238
         **:*:*::.. **. .  :****:* **** *:* :* :**:.** *:* ******.******* .:**:* :*** *::

                                            RdRpD
USUV     LIGRMEKRTWHGPKYEEDVNLGSGTRAVGKPQPHTNQEKIKARIQRLKEEYAATWHHDKDHPYRTWTYHGSYEVKPTGSA     320
WNV      LLGRMEKKTWKGPQFEEDVNLGSGTRAVGKPLLNSDTSKIKNRIERLKKEYSSTWHQDANHPYRTWNYHGSYEVKPTGSA     320
JEV      LLGRMDRTVWRGPKYEEDVNLGSGTRAVGKGEVHSNQEKIKKRIQKLKEEFATTWHKDPEHPYRTWTYHGSYEVKATGSA     320
DENV1    LLNRFTM-AHRKPTYERDVDLGAGTRHVAVEPEVANLDIIGQRIENIKNEHKSTWHYDEDNPYKTWAYHGSYEVKPSGSA     316
DENV2    LINRFTM-RYKKATYEPDVDLGSGTRNIGIESEIPNLDIIGKRIEKIKQEHETSWHYDQDHPYKTWAYHGSYETKQTGSA     317
         *:.*:     :   :* **:**:*** :.      : . *  **:.:*:*. ::** * ::**:** ******.* :***

USUV     SSLVNGVVRLMSKPWDAILNVTTMAMTDTTPFGQQRVFKEKVDTKAPEPPSGVREVMDETTNWLWAFLAREKKPRLCTRE     400
WNV      SSLVNGVVRLLSKPWDTITNVTTMAMTDTTPFGQQRVFKEKVDTKAPEPPEGVKYVLNETTNWLWAFLARDKKPRMCSRE     400
JEV      SSLVNGVVKLMSKPWDAIANVTTMAMTDTTPFGQQRVFKEKVDTKAPEPPAGAKEVLNETTNWLWAHLSREKRPRLCTKE     400
DENV1    SSMVNGVVRLLTKPWDVIPMVTQIAMTDTTPFGQQRVFKEKVDTRTPKAKRGTAQIMEVTARWLWGFLSRNKKPRICTRE     396
DENV2    SSMVNGVVRLLTKPWDVVPMVTQMAMTDTTPFGQQRVFKEKVDTRTQEPKEGTKKLMKITAEWLWKELGKKKTPRMCTRE     397
         **:*****:*::****.:  ** :*******************:: :   *.  ::. *:.***  *.:.* **:*::*

                                                                               F
USUV     EFKRKVNSNAALGAMFEEQNQWSSAREAVEDPRFWEMVDEERENHLKGECHTCIYNMMGKREKKLGEFGKAKGSRAIWFM     480
WNV      EFIGKVNSNAALGAMFEEQNQWKNAREAVEDPKFWEMVDEEREAHLRGECNTCIYNMMGKREKKPGEFGKAKGSRAIWFM     480
JEV      EFIKKVNSNAALGAVFAEQNQWSTAREAVDDPRFWEMVDEERENHLRGECHTCIYNMMGKREKKPGEFGKAKGSRAIWFM     480
DENV1    EFTRKVRSNAAIGAVFVDENQWNSAKEAVEDERFWDLVHRERELHKQGKCATCVYNMMGKREKKLGEFGKAKGSRAIWYM     476
DENV2    EFTRKVRSNAALGAIFTDENKWKSAREAVEDSRFWELVDKERNLHLEGKCETCVYNMMGKREKKLGEFGKAKGSRAIWYM     477
         **  **.****:**:* ::*:*..*:***:* :**::*..**: * .*:* **:********** *************:*

                                                             A
USUV     WLGARFLEFEALGFLNEDHWLGRKNSGGGVEGLGVQKLGYILREMSHHSGGKMYADDTAGWDTRITRADLDNEAKVLELM     540
WNV      WLGARFLEFEALGFLNEDHWLGRKNSGGGVEGLGLQKLGYILKEVGTKPGGKVYADDTAGWDTRITKADLENEAKVLELL     540
JEV      WLGARYLEFEALGFLNEDHWLSRENSGGGVEGSGVQKLGYILRDIAGKQGGKMYADDTAGWDTRITRTDLENEAKVLELL     540
DENV1    WLGARFLEFEALGFMNEDHWFSRENSLSGVEGEGLHKLGYILRDISKIPGGNMYADDTAGWDTRITEDDLQNEAKITDIM     536
DENV2    WLGARFLEFEALGFLNEDHWFSRENSLSGVEGEGLHKLGYILRDVSKKEGGAMYADDTAGWDTRITLEDLKNEEMVTNHM     537
         *****:********:*****:.*:** .**** *::******:::.   ** :************   **.**  : : :

                                                   B
USUV     EGEHRQLARAIIELTYKHKVVKVMRPGTDGKTVMDVISREDQRGSGQVVTYALNTFTNIAVQLIRLMEAEGVIGQEHLES     620
WNV      DGEHRRLARSIIELTYRHKVVKVMRPAADGKTVMDVISREDQRGSGQVVTYALNTFTNLAVQLVRMMEGEGVIGPDDVEK     620
JEV      DGEHRMLARAIIELTYRHKVVKVMRPAAEGKTVMDVISREDQRGSGQVVTYALNTFTNIAVQLVRLMEAEGVIGPQHLEQ     620
DENV1    EPEHALLATSIFKLTYQNKVVRVQRPAKNG-TVMDVISRRDQRGSGQVGTYGLNTFTNMEAQLIRQMESEGIFSPSELET     615
DENV2    EGEHKKLAEAIFKLTYQNKVVRVQRPTPRG-TVMDIISRRDQRGSGQVGTYGLNTFTNMEAQLIRQMEGEGVFKSIQHLT     616
         : **  ** :*::***::***:* **   * ****:***. ******** **.******: .**:* **.**::    .

                                       C                   D                   E
USUV     LPRKTKYAVRTWLFENGEERVTRMAVSGDDCVVKPLDDRFANALHFLNSMSKVRKDVPEWKPSSGWHDWQQVPFCSNHFQ     720
WNV      LGKGKGPKVRTWLFENGEERLSRMAVSGDDCVVKPLDDRFATSLHFLNAMSKVRKDIQEWKPSTGWYDWQQVPFCSNHFT     720
JEV      LPRKTKIAVRTWLFENGEERVTRMAISGDDCVVKPLDDRFATALHFLNAMSKVRKDIQEWKPSHGWHDWQQVPFCSNHFQ     720
DENV1    P--NLAERVLDWLKKHGTERLKRMAISGDDCVVKPIDDRFATALTALNDMGKVRKDIPQWEPSKGWNDWQQVPFCSHHFH     713
DENV2    I--TEEIAVQNWLARVGRERLSRMAISGDDCVVKPLDDRFASALTALNDMGKIRKDIQQWEPSRGWNDWTQVPFCSHHFH     714
                 *  ** . * **:.*****:*********:*****.:*  ** *.*:****: :*:** ** ** *******:**

           E                                                             Priming loop
USUV     ELIMKDGRTLVVPCRGQDELIGRARVSPGSGWNVRDTACLAKAYAQMWLLLYFHRRDLRLMANAICSAVPSNWVPTGRTS     800
WNV      ELIMKDGRTLVVPCRGQDELIGRARISPGAGWNVRDTACLAKSYAQMWLLLYFHRRDLRLMANAICSAVPANWVPTGRTT     800
JEV      EIVMKDGRSIVVPCRGQDELIGRARISPGAGWNVKDTACLAKAYAQMWLLLYFHRRDLRLMANAICSAVPVDWVPTGRTS     800
DENV1    QLIMKDGREIVVPCRNQDELVGRARVSQGAGWSLRETACLGKSYAQMWQLMYFHRRDLRLAANAICSAVPVDWVPTSRTT     793
DENV2    ELIMKDGRVLVVPCRNQDELIGRARISQGAGWSLRETACLGKSYAQMWSLMYFHRRDLRLAANAICSAVPSHWVPTSRTT     794
         :::****** :*****.****:****:* *:**.:::****.*:***** *:********* ********* .****.**:

          Priming loop
USUV     WSVHATGEWMTTDDMLEVWNKVWIQDNEWMLDKTPVQSWTDIPYTGKREDIWCGSLIGTRTRATWAENIYAAINQVRAII     880
WNV      WSIHAKGEWMTTEDMLAVWNRVWIEENEWMEDKTPVERWSDVPYSGKREDIWCGSLIGTRTRATWAENIHVAINQVRSVI     880
JEV      WSIHSKGEWMTTEDMLQVWNRVWIEENEWMMDKTPITSWTDVPYVGKREDIWCGSLIGTRSRATWAENIYAAINQVRAVI     880
DENV1    WSIHAHHQWMTTEDMLSVWNRVWIEENPWMEDKTHVSSWEDVPYLGKREDQWCGSLIGLTARATWATNIQVAINQVRRLI     873
DENV2    WSIHAKHEWMTTEDMLTVWNRVWIQENPWMEDKTPVESWEEIPYLGKREDQWCGSLIGLTSRATWAKNIQAAINQVRSLI     874
         **:*:  :****:*** ***:***::* ** *** :  *:::** ***** *******   ***** ** .******  *

USUV     GQEKYRDYMLSLRRYEEVNVQEDRVL 906
WNV      GEEKYVDYMSSLRRYEDTIVVEDTVL 906
JEV      GKENYVDYMTSLRRYEDVLIQEDRVI 906
DENV1    GNENYLDFMTSMKRFKNESDPEGALW 899
DENV2    GNEEYTDYMPSMKRFRREEEEAGVLW 900
         * :**:*:* *:* *::*:.  . :
```

Supplementary figure 3. Flavivirus NS5 amino acid sequence alignment. Amino acid sequence of the USUV, JEV, WNV, and DEND NS5 proteins were aligned with ClustalW software. Symbols meaning is as follows: An * (asterisk) indicates positions which have a single, fully conserved residue; a : (colon) indicates conservation between groups of strongly similar properties - scoring > 0.5 in the Gonnet PAM 250 matrix; a . (period) indicates conservation between groups of weakly similar properties - scoring =< 0.5 in the Gonnet PAM 250 matrix. Arrow indicate the starting for RdRpD. The minimal abNLS according to reference 14 (amino acid 372 to 408) is shown in bold red. The position of the triple alanine substitution mutant used in this study is indicated by an orange box. A-F and priming loop are polymerase domains.

Supplementary Figure 4

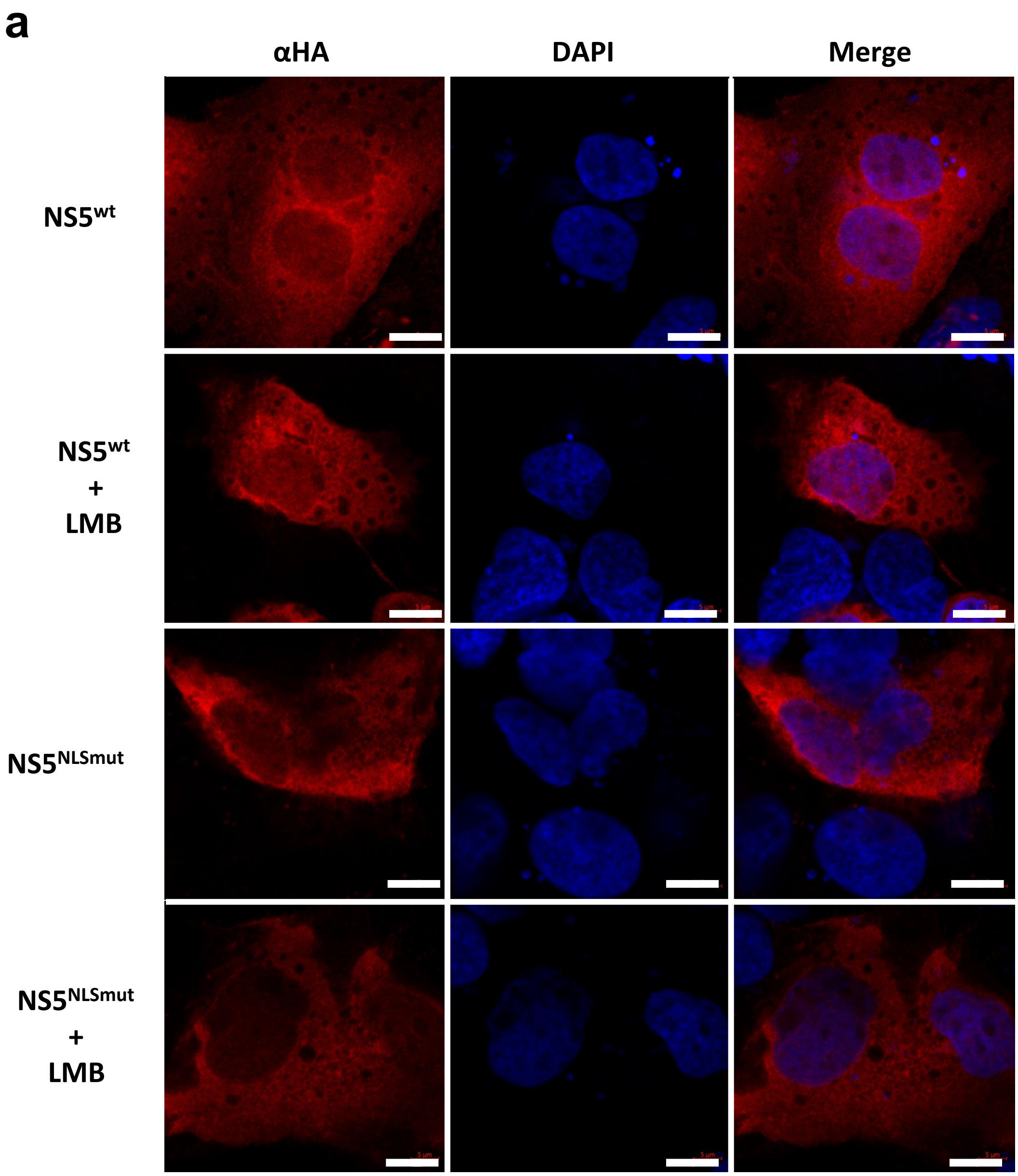

Supplementary Figure 4

b

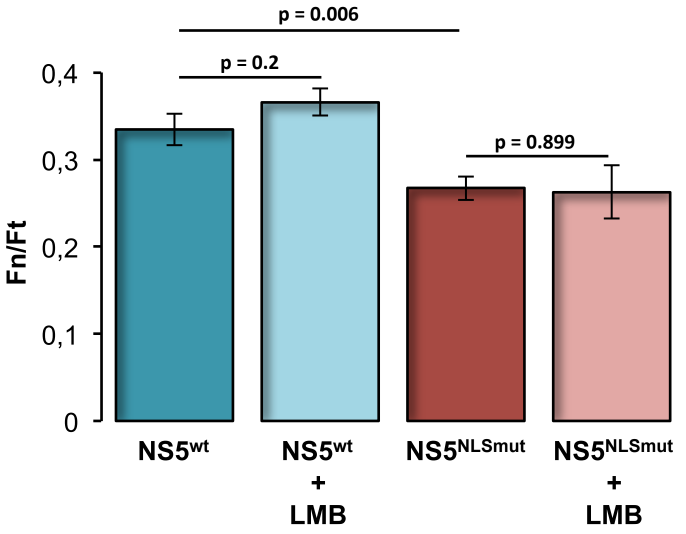

c

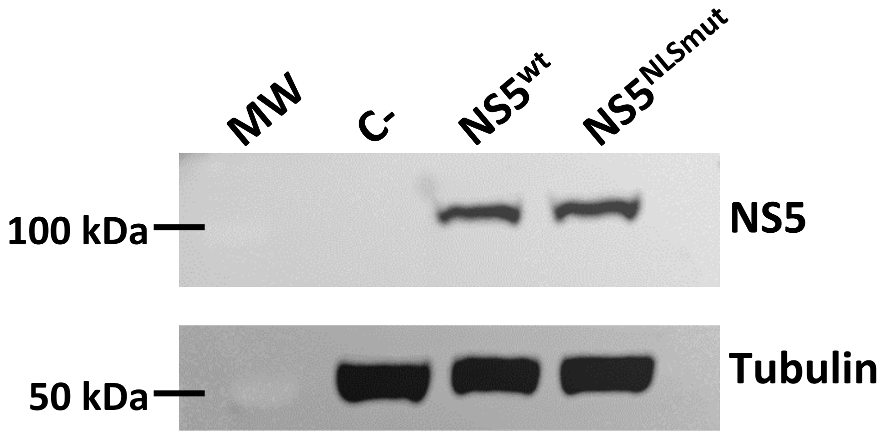

Supplementary figure 4. NLS role on subcellular localization of USUV NS5. a. Huh7.5 cells were transiently transfected with a plasmid encoding the full-length NS5 protein (pcDNA3-NS5-HA) or its corresponding mutant REK to AAA in the NLS sequence (pcDNA3-NS5^{NLSmut}-HA). When indicated, cells were treated with LMB 40 nM 24 hours post transfection. Cells were fixed for immunofluorescence 30 hours post transfection. Immunocytochemistry was performed using antibodies against HA (in red), detecting NS5 and NS5^{NLSmut}, and with DAPI (in blue), detecting cell nuclei. αHA panels correspond to immunostaining with anti-HA whereas DAPI panels correspond to cell nuclei staining. Merged images show an overlay with co-localization. Scale bars correspond to 5 µm. **b**. Mean nuclear fluorescence was quantified using the ZEISS software ZEN 2 (blue edition) and Fn/Ft determined. Twenty cells per sample were analyzed from independent duplicate experiments. Data represent the mean (± standard error of the mean, SEM). P-values are indicated above. **c**. Blot showing the total amount of NS5^{wt} and NS5^{NLSmut} proteins in Huh7.5. Tubulin is shown as a loading control.
